# Vicarious experiences of long COVID: A protection motivation theory analysis for vaccination intentions

**DOI:** 10.1016/j.jvacx.2023.100417

**Published:** 2023-12-07

**Authors:** Sarah Eitze, Philipp Sprengholz, Lars Korn, Parichehr Shamsrizi, Lisa Felgendreff, Cornelia Betsch

**Affiliations:** a Health Communication, Department of Implementation Research, Bernhard-Nocht-Institute for Tropical Medicine, Hamburg, Germany; b Institute for Planetary Health Behavior (IPB), University of Erfurt, Erfurt, Germany; c Institute of Psychology, University of Bamberg, Germany; d Hanover Center for Health Communication, Department of Journalism and Communication Research, Hanover University of Music, Drama, and Media, Hanover, Germany

**Keywords:** Protection motivation theory, Vaccination intentions, COVID-19, Long COVID, Health communication

## Abstract

**Context:**

Long COVID can appear as a severe late consequence (sequela) of a COVID-19 infection, leading to the inability to work or participate in social life for an unknown amount of time. To see friends or family struggling with long COVID might influence people’s risk perceptions, vaccine efficacy expectations, and self-efficacy perceptions to prevent COVID-19 and its consequences.

**Methods:**

In an online survey in August 2022, n = 989 German-speaking participants indicated whether they knew someone who suffered from long COVID illness. Four dimensions of protection motivation theory (PMT) were assessed afterwards, as well as vaccination intentions.

**Results:**

Multiple mediation analysis with participants who knew vs. didn't know someone with long COVID (n = 767) showed that knowing someone with long COVID was associated with higher perceived affective and cognitive risk of long COVID-19 as well as higher perceived vaccine efficacy. Self-efficacy, i.e., the ease to protect oneself against long COVID, was lower in participants who knew long-COVID patients. Indirect positive effects for response efficacy and affective risk suggest that vicarious experience with long COVID is associated with increased intentions to get a COVID-19 vaccine.

**Conclusion:**

The protection from long COVID through vaccination are relevant aspects for individual decisions and health communication.

## Introduction

For three years, the COVID-19 pandemic changed public and individual dimensions of health behavior [1]. Even though after only one year, several vaccines had been developed and approved, these vaccines have neither been universally available nor accepted [2] despite the fact COVID-19 can lead to an adverse late effect called long or post COVID [3]. In 2022, the US Centers for Disease Control and Prevention defined long COVID as a persistence or appearance of symptoms like fatigue, breathing difficulties, and cognitive dysfunctions that last 4 weeks or longer after a COVID-19 infection [4]. This study investigated whether vicarious experiences of long COVID —from observing it in the personal social environment—affects the intention to get vaccinated against COVID-19. Following protection motivation theory (PMT),[5] vicarious experiences of long COVID might influence the dimensions of coping appraisal and threat appraisal differently. Studies using the so-called sequelae approach [6] in health communication showed first promising results that sequelae such as long COVID are considered in vaccination decisions and decrease vaccine hesitancy.

Vaccine hesitancy refers to delayed acceptance or refusal of vaccines despite the availability of vaccination services [7]. Even before the COVID-19 vaccines were introduced in December 2020, several studies found reluctance to take the vaccine on all continents and in all risk groups [8]. A large-scale study across 23 countries found that within one year, the percentage of those who did not want to get vaccinated decreased from 28.5 % to 24.8 %. Hesitancy ranged from over 40 % in Russia, Poland, and Nigeria to under 10 % in China and Brazil [9]. A recent *meta*-analysis from the US also found up to 39 %, but within the country, there were large differences depending on political affiliation, ethnicity, and other demographic differences [10]. These studies emphasize that there is still a need for targeted health information to decrease vaccine hesitancy during the time of data collection and beyond. Furthermore, within the pandemic situation, evidence can be gathered regarding theories such as PMT. We can test whether vaccination decisions in a pandemic are based on the same dimensions as routine vaccination decisions.

Protection motivation theory proposes a set of dimensions that can be applied to understanding vaccine intentions and decisions [11]. This theory consists of two main dimensions: threat appraisal and coping appraisal [5]. In a threat appraisal, individuals assess the severity of the situation and examine how serious the situation is, while a coping appraisal encompasses how one responds to the situation. Threat appraisals include perceived cognitive and affective risk (e.g., of long COVID-19). Dimensions such as perceived vulnerability, perceived severity, and fear are included. Coping appraisals include response efficacy and self-efficacy. Response efficacy refers to the belief that the recommended action (e.g., vaccination) will be effective in reducing the threat, while self-efficacy refers to the belief that one can successfully prevent the undesired outcome (e.g., long COVID as a sequela of COVID-19) [5]. During the COVID-19 pandemic, several studies used the components of protection motivation theory to explain the underlying mechanisms of vaccine hesitancy. Dimensions of PMT explained 59 % of the variance in vaccination intentions in a UK sample of people between 50 and 64 years of age [12]. A study by Ansari-Moghaddam [13] identified response efficacy as the strongest predictor of vaccination intentions in Iran, followed by perceived self-efficacy and the severity of the disease.

### Research questions

At the time of the study, reliable scientific knowledge about the incidence (probability), severity, and duration of long COVID was unavailable. Long COVID’s most common symptoms include breathing difficulties and cognitive dysfunctions, such as fatigue, brain fog, or headaches. Often, depression and memory issues occur as well [14]. All of these symptoms can lead to the inability to work or participate in social life, an extremely severe consequence, especially when the duration of this consequence is unknown. Observing long COVID in a friend or relative could yield critical vicarious experiences that might result in higher risk perceptions—and, in the end, greater intentions to get vaccinated. To explore this, we focused on the following research questions (RQ):

*RQ1: Is knowing someone with long COVID (i.e., the vicarious experience of long COVID) related to higher perceived cognitive risk, which is in turn related to higher vaccination intentions?*

Especially because risk factors are unknown and treatment options are still rare, affective risk (i.e., the feeling of fear and worry) may increase with knowing someone with long COVID. Following PMT, this is related to higher protection motivation. Therefore, RQ2 asks:

*RQ2: Is knowing someone with long COVID related to higher affective risk, which is in turn related to higher vaccination intentions?*

Another important aspect of PMT is efficacy evaluation. Knowing someone with long COVID might influence self-efficacy, that is, lead to the feeling that one might not be able to protect oneself against long COVID. Low self-efficacy has been found to be associated with lower protection motivation in a *meta*-analytic review of the COVID-19 pandemic [15]. Therefore, RQ3 explores the relationship between vicarious experience, self-efficacy, and vaccination intention:

*RQ3: Is knowing someone with long COVID related to lower self-efficacy, which is in turn related to lower vaccination intentions?*

Seeing how relatives or friends experience long COVID might also influence the perceived response efficacy of the vaccine. By protecting against COVID-19 infections, the vaccine also protects against long COVID; that is, knowing about this should be related to greater perceived response efficacy. Therefore, the next research question focuses on this relationship:

*RQ4: Is knowing someone with long COVID related to higher response efficacy, which is in turn related to higher vaccination intentions?*

During the time of data collection, vaccination status varied widely; therefore, the intention to get vaccinated could mean several things: (a) unvaccinated people could get their first vaccination, (b) those with two shots could complete their immunization series (with a third vaccine), or (c) those with a completed series of three vaccinations could get a (fourth) shot with an updated vaccine to be protected against additional virus strains. Apart from knowing someone with long COVID, the four dimensions of protection motivation theory might still influence the intention to get vaccinated. Therefore, the last research question focuses on the general role of PMT constructs in vaccination intentions.

*RQ5: To what extent do the four dimensions of PMT explain differences in vaccination intentions?*

## Materials and methods

The data for this study were collected within the COVID-19 Snapshot Monitoring (COSMO). The COSMO-study assessed roughly 1,000 participants in weekly to fortnightly and later monthly online serial cross-sectional data collections. The monitoring project started in March 2020 and continued for a total of 70 waves until March 2023. Methodological preprints from the beginning of the survey are available [16] and were used to establish a general standard protocol by WHO Europe [17]. Data for this study were collected from 29 to 30 August 2022. A data collection company (Bilendi) invited and incentivized participants. The questionnaire included socio-demographic questions, monitoring questions about risk perceptions, trust in institutions, protective measures, worries, and policy acceptance measures as well as long COVID items regarding self-efficacy, response efficacy, and cognitive and affective risks. At the end of the survey, there were additional experimental studies on other topics that were reported elsewhere (in prep.). The items used for this study are now described in detail.

### Measures

*Vicarious long COVID experiences*. We used the question “Do you know someone who suffers from long COVID?” as a proxy for vicarious long-COVID experiences. Alongside this question, the definition of long COVID was provided (“Long COVID currently includes health complaints that persist beyond the acute illness phase of a SARS-CoV-2 infection of 4 weeks, but there might be new emerging symptoms as well.”; CDC, 2022). Answer options were (a) Yes, I know at least one person, (b) No, I don’t know anyone, (c) I’m not sure, or (d) I prefer not to answer.

*Cognitive risk.* The cognitive dimensions of risk were assessed as two dimensions [18] that were both answered on a 7-point scale (susceptibility: “What do you consider to be your own susceptibility to getting a long COVID disease?”; severity: “How severe would long COVID be for you?” from *harmless* to *very severe*). Cronbach’s alpha was *α* = 0.65.

*Affective risk perceptions.* We included three items regarding the affective risk for long COVID on 7-point semantic differentials: fear (“To me, long COVID feels … *not fear-inducing* to *fear-inducing*.”), worry (“To me, long COVID feels … *not at all worrying* to *very worrying*.”), and dominance of the topic (“To me, long COVID is … *something I rarely think about* to *something I think about all the time*.”). Cronbach’s alpha was *α* = 0.89.

*Efficacy measures.* Self-efficacy was assessed with one item (“For me, avoiding long COVID in the current situation is … *very hard* to *very easy*) on a 7-point scale. Response efficacy was also measured with one item using a 7-point scale (“The COVID-19 vaccine is effective against long COVID.” *totally disagree* to *totally agree*) [19], [20].

*Intention to get vaccinated.* The intention to get vaccinated was collected with one item on a 7-point scale (“If a vaccination is recommended and you had the opportunity to get vaccinated next week, would you take it?” *definitely not* to *definitely vaccinate*) [21].

*Sociodemographic variables and vaccination status.* In addition to age (collected as an open answer), gender (female/male/diverse), and education (low: up to 9 years; medium: 10 years without university qualification; high: 10 years or more with university qualification), all participants indicated how many COVID-19 vaccines they received (0–4).

### Sample

The sample of *n* = 998 participants was drawn to be quota representative for age and gender (crossed) and federal state residence so that the distributions on these variables reflected the distribution of the German population by the census [22]. The sample was slightly higher educated than the German population, as reflected by 56 % having at least a degree that qualifies for university. Of the sample, 10 % had never been vaccinated, and 16 % had had one or two doses but did not take a booster vaccine. Fifty-nine percent had been vaccinated a third time, and 15 % had taken a fourth vaccine against COVID-19. Of the participants, 26.6 % reported knowing someone with long COVID symptoms, 57.5 % did not know anyone with long COVID symptoms, 13.6 % were unsure, and 2.2 % refused to answer. Those using the last two categories were excluded from the mediation analyses, so that the final analysis sample was *n* = 839 (*n* = 265 knowing someone and *n* = 574 not knowing anyone).

### Statistical analyses

Our main dependent variable was protection motivation, measured as the intention to get vaccinated. Multiple mediation analyses [23] tested whether there were meaningful relationships between knowing someone with long COVID and vaccination intentions, mediated by cognitive risk (RQ1), affective risk (RQ2), self-efficacy (RQ3), and response efficacy (RQ4). Model 4 of the PROCESS macro [23] was chosen to include all four dimensions as equal mediators. We included all participants in one mediation analysis to reach sufficient statistical power [24]. Because there were three different choices based on previous behavior, namely, starting the immunization (first shot) versus completing their immunization (third shot) and continuing with an updated vaccine for the newest variants (fourth shot), we also ran separate multiple regressions, regressing each vaccination intention on sociodemographic variables and the four dimensions of protection motivation theory (cognitive risk, affective risk, self-efficacy, and response efficacy). The relationships between the dependent variables and the predictors were linear. Shapiro-Wilk tests indicated no normal distribution in vaccine intentions for any of the regressions, and heteroscedasticity was found for one regression (unvaccinated participants). There were no indicators of multicollinearity (all variance inflation factors (VIFs) between 1.02 and 2.33). The raw data, respective tests, and R Studio analysis scripts can be found here: https://osf.io/gvqd5/?view_only=ecfb174a1db840dfa66940a3f8d9bd1e.

## Results

**Fig. 1:**
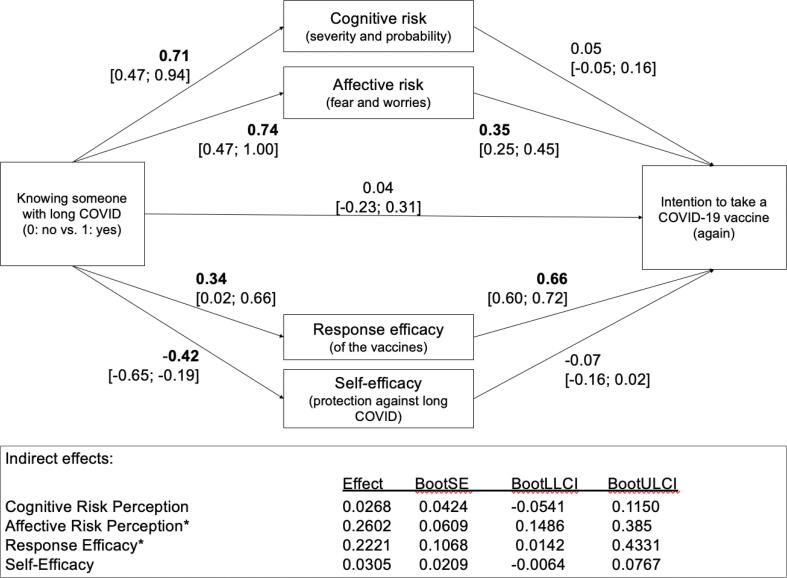
Mediation analysis for the relation between long COVID experience, dimensions of PMT, and the intention to vaccinate. *Note*. The figure shows the mediation analysis for the effect of knowing somebody with long COVID in the intention to get vaccinated via the four dimensions of protection motivation theory. Knowing somebody with long COVID (1) vs. not (0) is related to higher cognitive risk, higher affective risk (feeling fearful), and higher response efficacy for the vaccine. In this analysis, only people with a definite answer regarding long covid experiences were asked (n_no_ = 574; n_yes_ = 265), answers indicating uncertainty and missing answers were excluded.

The direct and indirect effects in the mediation analyses are shown in Fig. 1. Full mediation analysis results are presented in Supplementary Table 1. Knowing someone who suffers from long COVID was associated with all four dimensions of PMT: with higher cognitive and affective risk associated with long COVID, higher response efficacy (effectiveness of the vaccine to prevent long COVID), and lower self-efficacy (to be able to avoid a long COVID disease). Two of these four dimensions were also significantly related to vaccination intentions: higher affective risk and higher response efficacy were related to higher vaccine intentions, as expected. To answer the research questions, indirect effects are important. The indirect effects for cognitive risk (RQ1) and self-efficacy (RQ4) were not significant, as 95 % confidence intervals included 0 as a possible effect. The indirect effects for affective risk (RQ2) and response efficacy (RQ3) significantly differed from 0, indicating a positive relationship between vicarious experiences of long COVID and greater affective risk but also with higher response efficacy (the belief that vaccination can protect against long COVID) and, in turn, higher vaccination intentions.

Table 1 shows the results of the three regression analyses that examined (a) the predictors of intention to receive the first COVID-19 vaccine (unvaccinated participants), (b) the intention to receive the third vaccine (completing immunization), and (c) the intention to receive a fourth vaccine (to get an updated vaccine for the upcoming winter). In general, the results indicate that response efficacy—that is, the perception that the vaccine is effective in preventing long COVID—predicted all intentions to get vaccinated. The intention to get the first vaccination (*n* = 103 unvaccinated participants) was only predicted by response efficacy (*β* = 0.60, *p* < 0.001); neither cognitive (*β* = 0.21, *p* = 0.069) nor affective risk perception (*β* = −0.10, *p* = 0.333) were significant predictors. Self-efficacy showed no significant relationship either (*β* = 0.00, *p* = 0.953). For completing the immunization series (third vaccine, *n* = 156 participants vaccinated twice) and the fourth vaccination with an updated vaccine (*n* = 584 participants with three vaccinations), affective risk was a significant predictor (complete series: *β* = 0.19, *p* = 0.009; updated vaccine: *β* = 0.21, *p* < 0.001). Cognitive risk was not a significant predictor for either vaccination intention (complete series: *β* = 0.11, *p* = 0.122; updated vaccine: *β* = 0.02, *p* = 0.751) or self-efficacy (complete series*: β* = 0.01, *p* = 0.953; updated vaccine: *β* = 0.01, *p* = 0.260).

**Table 1:** Regression analyses for socio-demographics, risk perceptions and efficacy on the intention to vaccinate against COVID-19 for groups with different vaccination status.

	**Intention** **1st vaccine** **(unvaccinated)**			**Intention** **3rd vaccine** **(vaccinated twice)**			**Intention** **4th vaccine** **(vaccinated three times)**		
*Variables*	*std. Beta*	*std. CI*	*p*	*std. Beta*	*std. CI*	*p*	*std. Beta*	*std. CI*	*p*
Age	−0.11	−0.26 – 0.05	0.180	0.02	−0.11 – 0.14	0.794	0.09	0.01 – 0.16	**0.027**
Gender (female vs. male)	−0.12	−0.42 – 0.17	0.403	−0.18	−0.43 – 0.07	0.162	−0.04	−0.18 – 0.11	0.620
Education (high vs. low)	−0.01	−0.48 – 0.47	0.983	0.20	−0.28 – 0.68	0.403	0.19	−0.06 – 0.44	0.143
Education (medium vs. low)	−0.12	−0.61 – 0.37	0.629	0.18	−0.33 – 0.70	0.484	−0.03	−0.29 – 0.22	0.808
Affective risk (LC)	−0.10	−0.31 – 0.10	0.328	0.19	0.05 – 0.32	**0.009**	0.21	0.12 – 0.31	**<0.001**
Cognitive risk (LC)	0.20	−0.02 – 0.42	0.071	0.11	−0.03 – 0.24	0.122	0.02	−0.08 – 0.11	0.751
Response efficacy (LC)	0.60	0.42 – 0.78	**<0.001**	0.56	0.43 – 0.70	**<0.001**	0.44	0.36 – 0.51	**<0.001**
Self−efficacy (LC)	0.00	−0.15 – 0.16	0.953	0.01	−0.12 – 0.14	0.858	−0.04	−0.12 – 0.03	0.260

Observations (n)	103			156			584		
R^2^ / R^2^ adjusted	0.502 / 0.460			0.467 / 0.439			0.269 / 0.258		

*Note.* Regression analyses for the intention to vaccinate (a) for unvaccinated people receiving their first shot, (b) people who are vaccinated twice, completing their immunization, or (c) those vaccinated three times, expecting a dose with an updated vaccine. Standardized beta coefficients (std. Beta), 95% confidence intervals (CI), and sample size (observations) are reported. LC = long COVID.

Socio-demographic variables were largely irrelevant, except in the regression predicting the intention to get the updated vaccine, where higher age was associated with a higher intention (*β* = 0.09, *p* = 0.027). In conclusion, regarding RQ5, there are PMT dimensions with explanatory power (response efficacy) and without explanatory power (cognitive risk) when it comes to the COVID-19 vaccine intentions.

## Discussion

Long COVID is a severe late consequence of a COVID-19 infection. Its duration, severity, and risk factors have yet to be better understood. During the period of lacking reliable scientific information, hearing stories about or knowing people who (think they) suffer from the disease may affect how people think about long COVID and the vaccine—which is discussed to protect against long COVID [25]. Thus, knowing someone with long COVID might affect individual vaccination decisions. This work aimed to explore the effect of such vicarious experiences on vaccination decisions by focusing on their effects on protection motivation. In this survey, we found that knowing someone with long COVID symptoms was indeed related to increased affective risk perceptions for the disease as well as response efficacy for the vaccine, which was in turn associated with increased vaccination intentions. On the other hand, self-efficacy was decreased by the knowledge of someone with long COVID but not associated with differences in vaccination intentions. Among all factors, response efficacy was the strongest predictor of vaccination intention. This dimension was the only one relevant to the intention to get the first, third, or fourth vaccine. These findings and their implications must be contextualized.

Knowing someone with long COVID was also related to lower self-efficacy. The cause of this effect might be due to in-group favoritism. When we see long COVID in people who are close to us, we see long COVID in people who we may perceive to have a similar lifestyle and are in a similar environment to us. As we perceive a similarity to people who were not effective in preventing long COVID at all, we might also perceive ourselves as less effective in our protective measurements. This effect is consistent with social identity theory [26], social dominance theory, and system justification theory, which offer explanations for the dynamics of intergroup relationships. Because the risk factors for long COVID are unknown, the only way to prevent long COVID is by preventing infection. When self-efficacy is lower (i.e., when people report that they find it difficult to avoid an infection), preventive behaviors for infections tend to occur less often, such as physical distancing, testing, or mask-wearing [15]. Therefore, interventions should incorporate components that target self-efficacy. Enhancing self-efficacy can contribute significantly to the effectiveness of interventions by empowering individuals to take informed actions regarding their health [27]. The results further showed that response efficacy was related to intentions among people who were still unvaccinated. If future studies establish that multiple boosters do not significantly reduce the risk of long COVID or provide only marginal reduction, this information could significantly influence vaccine intention among the public—and especially among unvaccinated citizens. Consequently, vaccination campaign developers must be prepared to adapt their interventions accordingly in either direction. Interventions should promptly be adjusted to align with the evolving understanding of vaccine effectiveness and its relationship to long COVID risk.

Knowing someone with long COVID was associated with both higher cognitive and affective risk perceptions, but only the latter had a significant relationship to vaccination intentions. This is in line with previous research, where affective risk was more strongly related to vaccination intentions than cognitive risk perceptions [28], [29]. In general, long COVID information might increase vaccination intentions, especially when further studies consolidate that vaccines are effective against long COVID [30], [31]. Previous work has shown that pointing to the sequelae of infectious diseases can convince people of the severity of the disease and increase vaccination intentions. This sequelae approach [6] could be used to implement the current findings—always given that vaccination indeed lowers the risk of long COVID [31]. The sequelae approach has been shown to change relevant dimensions of health behavior affecting protection motivation in pandemic (as well as routine) vaccination decisions [6].

### Strengths and limitations

This study was conducted as an online survey, so the advantages and limitations of online studies apply here as well [32]. It is especially noteworthy that we were able to collect data from unvaccinated participants because of the anonymous online survey method. We can now better understand which factors explain differences in the intentions of beginners of immunization versus those who have to complete their immunization versus those who intend to get an updated vaccine. The explanatory power of response efficacy (vaccine efficacy) was more important in the regression for unvaccinated participants than in the regressions for already vaccinated participants. As a limitation, it is important to note that not all assumption tests for regression analyses were passed. Also, the data were collected in Germany, one of the so-called WEIRD countries (western educated industrialized, rich, and democratic) [33]. Generalizability to other contexts with lower vaccine uptake, higher systematic barriers toward vaccination, or lower general trust in authorities and institutions is therefore limited [34] and replicational studies in different contexts are recommended.

Another limitation is that stating to know someone with long COVID might refer to very different vicarious experiences. Hearing from a friend who is not able to leave the house might be different from seeing your partner experiencing breathing difficulties and brain fog. This might lead to individual differences in risk perceptions after vicarious experiences. In addition, we did not include items for objective long COVID knowledge. Future studies should test long COVID knowledge interventions to replicate these first results and see if risk perceptions are especially causally related to knowledge about long COVID versus vicarious experience—and whether noetic knowledge versus vicarious experience create different types of risk perceptions (e.g., affective vs. cognitive risk perceptions) and behavior. Furthermore, it should be evaluated whether low self-efficacy can be increased by evidence-based information and by a combination of other psychological strategies, such as cues-to-action for other protective measures [35] as proposed in the Health Belief Model [36]. Finally, all participants read the definition of long COVID. This might have led to a general increase in risk perceptions in all participants, regardless of their experiences. The effects are therefore rather conservative; among naïve participants the effect could be even stronger. Further replication of the results should take this into account.

Increasing long COVID knowledge with health communication might be additionally beneficial because campaigns might decrease the stigmatization of long COVID patients and lead to more people reaching out for appropriate medical treatment when they experience ongoing symptoms. In the long term, this could lead to a better understanding of long COVID disease and a significant reduction of disease burden.

## Conclusion

In this sample, 27 % of the participants already knew someone with long COVID symptoms. It will be important to provide evidence-based information about this possible consequence of COVID-19 because this number will likely increase as infections persist, and reinfections occur. Knowing someone with long COVID symptoms increases risk perceptions and vaccine efficacy perceptions but lowers self-efficacy perceptions to prevent a long COVID disease. Apart from vicarious long COVID experiences, the dimensions of protection motivation theory differ in their predictive values for different vaccination decisions. Response efficacy is the only dimension that is a reliable predictor in all vaccination decisions, from starting the immunization (uptake of the first vaccine) to completing the immunization (with a third vaccine) to the demand for an updated vaccine (as a fourth shot). Conclusively, health interventions might focus on arguments from different dimensions when addressing vaccinated versus unvaccinated target groups.

## Consent statement

All participants gave informed consent prior to study participation. They were informed that data will be published anonymously and that they can terminate the questionnaire at any time to withdraw their consent.

## Ethics approval

Ethic approval was given by the ethical advisory board of the University of Erfurt (#20220525).

## Funding

This research was funded by the German Research Foundation, BE3970/11–1, 12–1; Federal Centre for Health Education, Robert Koch-Institute, Leibniz Centre for Psychological Information and Documentation, the Klaus Tschirra Stiftung , and the University of Erfurt (no funding numbers). The authors alone are responsible for the views expressed in this manuscript; they do not necessarily represent the views, decisions, or policies of the institutions with which the authors are affiliated. The funding source did not influence the study's design or the analysis of the results. All authors were involved in the development of the manuscript. The authors declare no conflict of interest.

## Declaration of competing interest

The authors declare the following financial interests/personal relationships which may be considered as potential competing interests: Prof Cornelia Betsch reports financial support was provided by Robert Koch Institute. Prof Cornelia Betsch reports financial support was provided by Federal Centre for Health Education. Prof Cornelia Betsch reports financial support was provided by German Research Foundation.

## Data Availability

The data and analysis code is available via an OSF link that is included in the manuscript.
