# Supplementary material for: Vicarious experiences of long COVID: A protection motivation theory analysis for vaccination intentions
Source: Vaccine X. 2023 Dec 7;16:100417. doi: 10.1016/j.jvacx.2023.100417 (PMC10772280; doi:10.1016/j.jvacx.2023.100417)
Supplement: Supplementary data 1 [file mmc1.docx]

********************* PROCESS for R Version 4.0.2 *********************

Written by Andrew F. Hayes, Ph.D. www.afhayes.com

Documentation available in Hayes (2022). www.guilford.com/p/hayes3

***********************************************************************

Model : 4

Y : VACC_INTENTION

X : LC_FREQ_P_R

M1 : LC_AFF_RISK

M2 : LC_COG_RISK

M3 : SE_LC

M4 : RE_LC

Sample size: 767

Custom seed: 654321

***********************************************************************

Outcome Variable: LC_AFF_RISK

Model Summary:

R R-sq MSE F df1 df2 p

0.1923 0.0370 2.8618 29.3756 1.0000 765.0000 0.0000

Model:

coeff se t p LLCI ULCI

constant 3.5761 0.0720 49.6659 0.0000 3.4347 3.7174

LC_FREQ_P_R 0.7371 0.1360 5.4199 0.0000 0.4701 1.0041

***********************************************************************

Outcome Variable: LC_COG_RISK

Model Summary:

R R-sq MSE F df1 df2 p

0.2118 0.0449 2.1385 35.9291 1.0000 765.0000 0.0000

Model:

coeff se t p LLCI ULCI

constant 3.4837 0.0622 55.9696 0.0000 3.3615 3.6059

LC_FREQ_P_R 0.7047 0.1176 5.9941 0.0000 0.4739 0.9355

***********************************************************************

Outcome Variable: SE_LC

Model Summary:

R R-sq MSE F df1 df2 p

0.1274 0.0162 2.1650 12.6135 1.0000 765.0000 0.0004

Model:

coeff se t p LLCI ULCI

constant 4.6341 0.0626 73.9947 0.0000 4.5111 4.7570

LC_FREQ_P_R -0.4201 0.1183 -3.5515 0.0004 -0.6523 -0.1879

***********************************************************************

Outcome Variable: RE_LC

Model Summary:

R R-sq MSE F df1 df2 p

0.0744 0.0055 4.1128 4.2535 1.0000 765.0000 0.0395

Model:

coeff se t p LLCI ULCI

constant 4.2917 0.0863 49.7196 0.0000 4.1222 4.4611

LC_FREQ_P_R 0.3362 0.1630 2.0624 0.0395 0.0162 0.6563

***********************************************************************

Outcome Variable: VACC_INTENTION

Model Summary:

R R-sq MSE F df1 df2 p

0.7038 0.4953 2.7601 149.3763 5.0000 761.0000 0.0000

Model:

coeff se t p LLCI ULCI

constant 0.7679 0.3279 2.3415 0.0195 0.1241 1.4117

LC_FREQ_P_R 0.0364 0.1372 0.2656 0.7906 -0.2329 0.3058

LC_AFF_RISK 0.3530 0.0478 7.3820 0.0000 0.2591 0.4468

LC_COG_RISK 0.0481 0.0539 0.7070 0.4798 -0.0677 0.1638

SE_LC -0.0725 0.0449 -1.6166 0.1064 -0.1606 0.0155

RE_LC 0.6605 0.0304 21.6910 0.0000 0.6007 0.7202

***********************************************************************

Bootstrapping progress:

|>>>>>>>>>>>>>>>>>>>>>>>>>>>>>>>>>>>>>>>>>>>>>>>>>>>>>>>>>>>>>>| 100%

**************** DIRECT AND INDIRECT EFFECTS OF X ON Y ****************

Direct effect of X on Y:

effect se t p LLCI ULCI

0.0364 0.1372 0.2656 0.7906 -0.2329 0.3058

Indirect effect(s) of X on Y:

Effect BootSE BootLLCI BootULCI

TOTAL 0.5395 0.1301 0.2863 0.7920

LC_AFF_RISK 0.2602 0.0609 0.1486 0.3852

LC_COG_RISK 0.0268 0.0424 -0.0541 0.1150

SE_LC 0.0305 0.0209 -0.0064 0.0767

RE_LC 0.2221 0.1068 0.0142 0.4331

******************** ANALYSIS NOTES AND ERRORS ************************

Level of confidence for all confidence intervals in output: 95

Number of bootstraps for percentile bootstrap confidence intervals: 5000
